# Hemisensory syndrome: Hyperacute symptom onset and age differentiates ischemic stroke from other aetiologies

**DOI:** 10.1186/s12883-021-02206-8

**Published:** 2021-04-27

**Authors:** Pei Xuan Koh, Joanna Ti, Seyed Ehsan Saffari, Zhen Yu Isis Claire Lim, Tianming Tu

**Affiliations:** 1 National Neuroscience Institute, 11 Jalan Tan Tock Seng, Singapore, 308433 Singapore; 2 Duke-National University of Singapore (NUS) Medical School, Singapore, Singapore

**Keywords:** Hemisensory syndrome, Ischemic stroke, Numbness, Magnetic resonance imaging

## Abstract

**Background:**

An important cause of hemisensory syndrome is ischemic stroke. However, the diagnostic yield of neuroradiological imaging on hemisensory syndrome is low. Therefore, we aim to describe patients hospitalized with isolated hemisensory syndrome, and to identify clinical features associated with an aetiology of ischemic stroke.

**Methods:**

We performed a single centre retrospective observation study, identifying patients who were hospitalised with hemisensory syndrome from October 2015 to March 2016, and whom underwent a magnetic resonance imaging (MRI) brain during the admission. Ischemic stroke was defined as the presence of restricted diffusion-weighted image on the MRI brain. Clinical information was analysed and compared between patients with and without stroke seen on MRI brain.

**Results:**

79 patients, 36 (45.6%) males and 43 (54.4%) females, aged between 30 to 87 years (mean 54), were included in the final analysis. 18 (22.8%) patients were identified to have an acute ischemic stroke. Clinical features associated with ischemic stroke in hospitalised patients with hemisensory syndrome include symptom onset of ≤24 h at presentation (odds ratio 31.4, 95% CI 3.89–254.4), advanced age (odds ratio 1.14, CI 1.05–1.25) and smoking (odds ratio 7.35, 95% CI 1.20–45).

**Conclusion:**

Older patients, with a history of smoking, and who present with an acute onset of symptoms, are more likely to have ischemic stroke as the cause of their hemisensory syndrome.

**Supplementary Information:**

The online version contains supplementary material available at 10.1186/s12883-021-02206-8.

## Background

The definition of hemisensory syndrome is variable, but essentially describes a feeling of altered sensation on one side of the body [1, 2]. A complete hemisensory syndrome [3, 4] would involve the entire face, arm and leg, with or without trunk involvement, on the same side in the absence of weakness, homonymous hemianopia, aphasia, agnosia, and apraxia. An incomplete hemisensory syndrome includes several clinical variants such as the cheiro-oral-crural syndrome, cheiro-oral syndrome, and isolated oral syndrome [3, 5].

An important neurological cause of hemisensory syndrome is pure sensory strokes, the most common infarct location being the thalamus [3, 4, 6, 7]. Other infarct locations include the internal capsule, parietal lobe [8], corona radiata and pons [3, 6]. Anatomic-clinical correlations have been derived from studies of pure sensory strokes, as well as thalamic strokes. In particular, the ventral posterior nuclei within the thalamus are reciprocally interconnected with the primary somatosensory cortices [9, 10]. An infarct within the thalamus or its thalamocortical projections can thus result in contralateral hemianesthesia. As the somatotopy of these nuclei is precise, thalamic strokes can produce focal sensory deficits in the affected regions, clinically manifesting as an incomplete hemisensory syndrome. For pure sensory syndromes due to capsular or corona radiata strokes, previous studies have suggested that the lesion should occupy the posterior quarter of the posterior limb of the internal capsule, where sensory tracts without motor fibres are believed to be located [6, 11].

In clinical practice, however, majority of patients presenting with hemisensory symptoms do not have a cerebrovascular event [1]. Following extensive investigative efforts, alternative diagnoses commonly made in patients with hemisensory syndromes include migraine [4, 12], major depression, and generalized anxiety disorder [1]. There is therefore a frequent clinical dilemma in differentiating a stroke vs non-stroke etiology in patients presenting with hemisensory syndrome. Attempts have been made to describe signs of functional sensory symptoms, such as “midline splitting”, or the exact splitting of sensation in the midline [13–15]. These are however non-specific, and even found to occur in thalamic strokes [2].

Therefore, we aim to describe the prevalence of ischemic stroke and characteristics of patients admitted to neurology with isolated hemisensory syndrome. We also aim to identify the specific clinical features which may point towards an ischemic etiology in patients with hemisensory syndrome.

## Methods

This study is a retrospective case-control study conducted at a single comprehensive stroke center (Tan Tock Seng Hospital, Singapore), on consecutive hospitalized patients between 1 October 2015 to 31 March 2016. Inclusion criteria: (1) isolated complete or incomplete hemisensory syndrome, regardless of the duration of symptoms; (2) brain MRI (magnetic resonance imaging) performed within the same admission. Exclusion criteria: (1) bilateral sensory symptoms; (2) concomitant focal neurological symptoms and signs such as diplopia, dysarthria, dysphagia, focal weakness or ataxia; (3) contraindication to brain MRI.

All patients had a physical examination performed by a trained neurologist. This included an evaluation of cranial nerve function, as well as sensory, motor and cerebellar examination. All subjects underwent basic investigations that included a complete blood count, renal panel and electrocardiogram. Brain MRI was performed in all patients within 72 h of presentation. In addition, patients with stroke had their NIHSS (National Institutes of Health Stroke Scale) recorded, and also underwent further investigations which included doppler ultrasound of the carotid arteries, transthoracic echocardiogram, and Holter monitoring (24–72 h duration). Based on these investigations, the mechanism of stroke was categorized using the TOAST (Trial of Org 10,172 in Acute Stroke Treatment) classification. Other investigations that were performed at the discretion of the managing neurologist included MRI of the cervical spine, nerve conduction studies and electromyography, and rarely CSF (cerebrospinal fluid) analysis or EEG (electroencephalography).

Available clinical information was retrieved through the hospital electronic medical records. Information collected included patient demographics, medical history, and details of the symptom presentation. The presence of traditional ischemic stroke risk factors, such as diabetes mellitus, hypertension, hyperlipidemia, smoking and atrial fibrillation, were also recorded. A previous study had also described migraine and psychiatric disorders (depression, anxiety disorder, schizophrenia) [1] as concurrent medical history in patient’s presenting with hemisensory syndrome, thus these were also included in our data collection.

Abnormal sensory symptoms were characterised into two categories: positive and negative [16]. Positive symptoms were characterised as presence of increased sensory symptoms, described as tingling (or pins and needles), pricking, tightening or burning. This may also be described as paraesthesia or dysesthesia [4]. Negative sensory symptoms were characterized by diminished or absent sensation. We also characterized the patients’ descriptions of their onset of symptoms and divided them into those with acute (≤24 h) versus subacute (> 24 h) symptom onset. This was calculated from the time of symptom onset to the time of presentation to the emergency department. All clinical information was retrieved blinded to the neuroimaging findings.

Cases of confirmed acute ischemic stroke were defined as subjects whose MRI brain performed during the hospitalization showed a lesion demonstrating restricted diffusion. The infarct locations in the patients with restricted diffusion were recorded. Patients who had no restricted diffusion on MRI brain were classified as “non-stroke” (controls). The final diagnoses made by the attending physicians were recorded.

### Imaging

All MRI brain and cervical spine studies were performed on one of the three MRI scanners in our center. Detailed technical information on the MRI scanners and sequences are available in the Supplementary data (Supplementary File 1). An independent neuroradiologist analyzed the images obtained and the presence of restriction of the diffusion weighted images (DWI) with corresponding attenuation of apparent diffusion co-efficient (ADC) were considered as neuroimaging evidence of ischemic stroke.

### Statistical analysis

Demographics and baseline clinical features were reported as mean ± standard deviation and frequency (percentage) for continuous and categorical variables, and compared between the two groups (stroke vs non-stroke) using independent two-sample t-test and Fisher’s exact test, respectively. Univariate logistic regression analysis was conducted to investigate the association between demographic and clinical parameters with stroke outcome, and odds ratio (OR) along with 95% confidence interval (95% CI) were reported. Multivariable logistic regression analysis was performed to adjust the association results for the selected variables via stepwise variable selection approach (including age, smoking, onset ≤24 h, presence of positive symptoms). Statistical significance was set at *p* < 0.05. Data analysis was performed in SAS version 9.4 for Windows (SAS Institute Inc., Cary, NC, USA).

## Results

From October 2015 to March 2016, a total of 2609 patients were admitted to the Neurology department at Tan Tock Seng Hospital. The study team identified 81 patients presenting with a hemisensory syndrome. The NIHSS score of these patients ranged from 0 to 1 (IQR 0–1). Out of the 81 patients, 2 were excluded from the study analysis as they did not receive an MRI brain during the hospitalisation. Amongst the 79 patients included in the final analysis, 18 patients (22.8%) were identified to have an acute ischemic stroke, as defined by the presence of a restricted DWI lesion on MRI, while 61 did not (77.2%).

Patients with stroke were significantly older (mean age 62.8 ± 8.7 years), than the patients without stroke (mean age 51.5 ± 11.7 years) (Table 1). There were also significantly more smokers (44.4%) in the stroke group compared to the non-stroke group (14.8%). The majority of patients with stroke had described a symptom onset of ≤24 h at presentation (88.9%), and all of them described the presence of negative sensory symptoms. Significantly more patients in the non-stroke group presented with positive sensory symptoms (55.7%) compared to the stroke group (11.1%). The presence of psychiatric history and other vascular risk factors such as diabetes mellitus, hypertension, hyperlipidaemia and atrial fibrillation, were not different between the cases and controls.

**Table 1:** Clinical characteristics of study subjects

Parameter	Stroke (*n* = 18)	Non-stroke (*n* = 61)	*p* value**
Age (years)	62.8 ± 8.7*	51.5 ± 11.7	<.001
Sex			0.180
Male	11 (61.1)	25 (41.0)	
Female	7 (38.9)	36 (59.0)	
Race			0.283
Chinese	18 (100)	48 (78.7)	
Malay	0 (0)	5 (8.2)	
Indian	0 (0)	7 (11.5)	
Others	0 (0)	1 (1.6)	
Diabetes Mellitus	5 (27.8)	6 (9.8)	0.113
Hypertension	11 (61.1)	21 (34.4)	0.057
Hyperlipidaemia	12 (66.7)	28 (45.9)	0.180
Smoking	8 (44.4)	9 (14.8)	0.018
Atrial Fibrillation	1 (5.6)	2 (3.3)	0.55
Schizophrenia	1 (5.56)	0 (0)	0.228
Depression	0 (0)	5 (8.2)	0.583
Anxiety disorder	1 (5.6)	3 (4.9)	1.00
Migraine	0 (0)	7 (11.48)	0.341
First episode	17 (94.4)	50 (82.0)	0.278
Onset ≤24 h	16 (88.9)	20 (32.8)	<.001
Presence of positive symptoms	2 (11.1)	34 (55.7)	<.001
Presence of negative symptoms	18 (100)	41 (67.2)	0.004

* Continuous variables reported as means ± standard deviation; Categorical variables reported as frequency (percent)

** Independent two-sample t-test and Fisher’s exact test for continuous and categorical variables

In patients with hemisensory syndrome, for every one-year increase in age, the chance of an acute ischemic stroke being the underlying aetiology increased by 9% (unadjusted odds ratio 1.09, 95% CI 1.03–1.16) (Table 2). Smokers were also 4.5 times more likely to have stroke as a cause of hemisensory syndrome compared to non-smokers (unadjusted odds ratio 4.5, 95% CI 1.4–14.3). The presence of positive symptoms decreased the chance of stroke as a cause of hemisensory syndrome by 88% (unadjusted odds ratio 0.12, 95% CI 0.03–0.51). An acute onset of symptoms (≤24 h) however, increased this by 13.4 times (unadjusted odds ratio 13.4, 95% CI 3.14–56.9).

**Table 2:** Association analysis of demographics and clinical features with stroke (vs non-stroke) outcome

Parameter	Univariate^*^		Multivariable^**^	
	Un-Adjusted OR (95% CI)	*p* value	Adjusted OR (95% CI)	*p* value
Age	1.09 (1.03, 1.16)	0.002	1.14 (1.05, 1.25)	0.003
Sex (Female vs male)	0.46 (0.16, 1.32)	0.147	–	–
Race (Chinese vs non-Chinese)	10.3 (0.52, 203)	0.125	–	–
Diabetes Mellitus	3.48 (0.92, 13.1)	0.066	–	–
Hypertension	2.89 (0.99, 8.44)	0.052	–	–
Hyperlipidaemia	2.26 (0.76, 6.69)	0.141	–	–
Smoking	4.47 (1.40, 14.3)	0.012	7.35 (1.20,45)	0.0310
Atrial Fibrillation	2.04 (0.19, 22.5)	0.560	–	–
Schizophrenia	10.4 (0.11, 983)	0.312	–	–
Depression	0.28 (0.01, 6.91)	0.435	–	–
Anxiety disorder	1.43 (0.16, 13.0)	0.749	–	–
Migraine	0.20 (0.01, 4.39)	0.304	–	–
First episode	2.66 (0.42, 16.9)	0.302	–	–
Onset ≤24 h	13.4 (3.14, 56.9)	< 0.001	31.4 (3.89, 254.4)	0.001
Presence of positive symptoms	0.12 (0.03, 0.51)	0.004	0.22 (0.04, 1.33)	0.099
Presence of negative symptoms	18.3 (0.98, 341)	0.0517	–	–

^*^ Univariate logistic regression analysis

^**^ Multivariable logistic regression analysis, stepwise variable selection approach

*OR* Odds Ratio; *CI* Confidence Interval

Age, smoking, and symptom onset ≤24 h remained significant at *p* < 0.1 after stepwise variable selection criteria in multivariate logistic regression analysis (Table 2). The presence of positive symptoms was no longer associated with a non-stroke cause of hemisensory syndrome (adjusted odds ratio 0.22, 95% CI 0.04–1.33). On the other hand, increased age, smoking and an acute onset of hemisensory symptoms at presentation remained significantly associated with ischemic stroke after adjustment, suggesting a strong likelihood of underlying stroke.

**Fig. 1:**
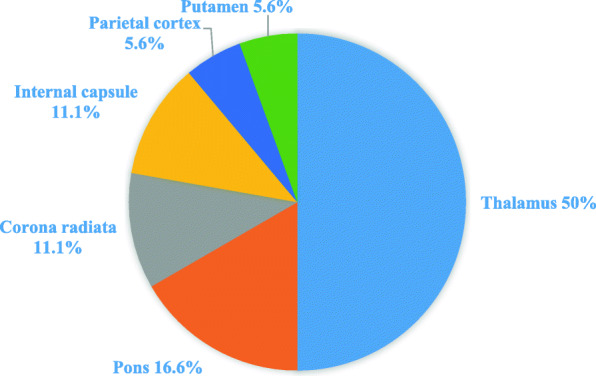
Pie chart showing the proportion of the various infarct locations in study cases

Amongst the patients with strokes, 50% of them had infarcts located in the thalamus (Fig. 1). The infarct locations included the pons, corona radiata, internal capsule, parietal cortex and putamen. The mechanism of stroke was classified as small vessel disease in 56% of patients, large vessel disease in 28%, undetermined aetiology in 11%, and cardioembolic in 5%. On discharge, majority of the patients had an NIHSS of 0 to 2; only one patient progressed to develop mild unilateral weakness, and was discharged with an NIHSS of 5.

Majority of the non-stroke patients did not have a specific diagnosis after investigations and were labelled as non-specific numbness or hemisensory syndrome (47.53%). In the remaining patients, the hemisensory symptoms were attributed to cervical radiculopathy in 27.87%, migraine in 8.2% and transient ischemic attack (TIA) in another 8.2%. Other rarer diagnoses made included meralgia paraesthetica, neuropathic pain, and panic attacks.

## Discussion

Conflicting data exist for the true incidence of ischemic strokes in hemisensory syndromes. A study of patients presenting to the outpatient or emergency department with hemisensory syndrome revealed an overall benign prognosis after a follow-up period of up to 16 months [1]. In this prospective study of 34 patients, only one patient (3%) was diagnosed with ischemic stroke after substantial diagnostic procedures. The studied population was predominantly female (74%), with more than 60% describing positive symptoms such as tingling, and an average symptom onset of 1.6 days prior to presentation [1]. On the contrary, in a recent study analyzing 1028 patients presenting to a stroke neurologist with minor neurologic events, 13.5% were found to have a DWI positive lesion on MRI brain scan. This study included 482 (46.9%) patients with isolated sensory symptoms [17].

The prevalence of radiologically confirmed ischemic stroke in hospitalized patients presenting to a Neurology unit with hemisensory symptoms was 22.8% in our study. We postulate that the relatively high incidence of ischemic stroke seen in our study population could be related to two main factors. Firstly, patients with hemisensory symptoms were included regardless of the duration of their symptoms. Previous pathological studies have also found lacunar infarcts in the thalamus on post-mortem in patients with TIA symptoms lasting ≤15 min [4]. This, together with a more recent study identifying DWI positive lesions in 13.5% of patients with transient neurological symptoms lasting < 5 min, suggest that the duration of symptoms alone may not help to distinguish a stroke versus non-stroke etiology of sensory symptoms. In addition, our patient population would have had an initial evaluation by a general or emergency physician prior to referral to the inpatient Neurology unit.

The diagnosis of ischemic stroke will alter subsequent clinical management, and secondary stroke prevention potentially may prevent further ischemia in this group of patients. Hence, the identification of high-risk clinical features that points towards ischemia, will be useful in prioritising neuroimaging in this group of patients. Our study suggests that older patients, with a history of smoking, and who present within 24 h of onset of hemisensory symptoms, are more likely to have stroke as the cause of their hemisensory syndrome. Although this may be clinically intuitive to most physicians, our study is the first to systematically analyse these clinical characteristics and their utility in predicting radiologically confirmed ischemic stroke as an underlying aetiology. These clinical features may therefore be helpful in triaging patients in the emergency department on the need for further imaging or observation.

In our study, the presence of positive symptoms displayed an initial trend towards a non-stroke aetiology being the cause of hemisensory syndrome. However, this characteristic did not reach statistical significance after multi-variable analysis. The most likely reason for this finding is that the description of sensory symptoms can be subject to physician interpretation. However, we feel that this part of the patient’s history may still be a valuable decision-making tool when used in conjunction with other clinical and demographic data.

The infarct locations for patients with strokes in our study were similar to previous localisation on pure sensory stroke [3, 6]. In concordance with previous literature [3], the main mechanism of stroke is small vessel disease, with a smaller proportion of patients diagnosed to have underlying large vessel atherosclerosis [3]. In the group of patients without stroke, other investigations were performed based on the history and examination to elucidate the cause of the sensory symptoms. In spite of this, a significant proportion of patients do not receive a specific diagnosis on discharge, and instead receive plans for follow-up visits in the Neurology outpatient clinic. Based on a previous study, majority of patients with hemisensory symptoms turn out to have a benign prognosis, and up to 80% of patients are symptom-free on subsequent reviews [1]. However, the need for subsequent reviews to monitor for progression or relapsing-remitting symptoms cannot be discounted as sensory symptoms may occasionally herald more sinister conditions such as demyelinating disease [18]. The common specific diagnoses subsequently conferred in the non-stroke group include cervical radiculopathy and migraine-related symptoms. This was similar to other authors [1, 4] who investigated sensory syndromes. Interestingly, 8.2% of patients with no DWI lesion on MRI were diagnosed to have a TIA. These patients were likely to have received antiplatelets and investigated in a similar manner to the patients with stroke. Future larger prospective studies may be useful to elucidate the clinical characteristics of patients labelled as TIA, to decide what influences the attending physicians’ decisions in labelling a hemisensory syndrome as due to a TIA. Moreover, the long-term outcomes of hemisensory syndrome patients diagnosed with TIA can be compared to a similar control group.

The main limitation of this study includes its relatively small sample size, which may result in the underrepresentation of individual vascular risk factors, and specific psychiatric history, thereby influencing the association with the outcome of interest. Nevertheless, this does not negate the influence of age, smoking and recent onset of sensory symptoms as the main 3 factors associated with a radiologically positive ischemic stroke. The retrospective nature of the study could have resulted in selection bias, whereby managing physicians did not choose to perform MRI in their patients, and hence excluded this group of individuals from this study.

From previous literature, hemisensory symptoms appear to be more prevalent amongst patients with underlying depression or anxiety [1]. In our study, the medical history and diagnoses of the patients were collected from electronic medical health records. As such, another limitation includes the inability to systematically assess the mental health of our patient population. Future prospective studies addressing these group of patients may consider using standardised questionnaires to assess for depression. In addition, subjective clinical recording of the sensory symptoms by the attending physicians may have influenced the classification of positive and negative symptoms. Lastly, this study is conducted on hospitalised patients in a single center. Thus, confirmation of the reported findings by international multi-centered studies is necessary for the results to be generalisable to other ethnic populations in various settings.

## Conclusion

In conclusion, a small but significant number of patients who present with hemisensory symptoms turned out to have positive DWI lesions on MRI brain. These patients were older, were smokers and had onset less than 24 h from presentation. It is important to keep these clinical factors in mind when deciding to perform neuroimaging for patients who present with hemisensory symptoms, as it may change the clinical management of these patients significantly.

## Supplementary Information

**Additional file 1.** Technical details of MRI scanners and parameters of specific MRI sequences.

## Data Availability

The datasets analysed during the current study are available from the corresponding author on reasonable request.

## Abbreviations

ADC: Apparent diffusion co-efficient
CI: Confidence interval
CSF: Cerebrospinal fluid
DWI: Diffusion weighted images
EEG: Electroencephalography
GRE: Gradient Echo
Min: Minute
MRI: Magnetic resonance imaging
MS: Milliseconds
NIHSS: National Institutes of Health Stroke Scale
OR: Odds ratio
S: Seconds
STIR: Short tau inversion recovery
T: Tesla
TE: Echo time
TIA: Transient ischemic attack
TOAST: Trial of Org 10,172 in Acute Stroke Treatment
TR: Repetition time
