# Supplementary material for: Hemisensory syndrome: Hyperacute symptom onset and age differentiates ischemic stroke from other aetiologies
Source: BMC Neurol. 2021 Apr 27;21:179. doi: 10.1186/s12883-021-02206-8 (PMC8077773; doi:10.1186/s12883-021-02206-8)
Supplement: Supplementary file 1 — Additional file 1. Technical details of MRI scanners and parameters of specific MRI sequences. [file 12883_2021_2206_MOESM1_ESM.docx]

Supplementary file 1: Technical details of MRI scanners and parameters of specific MRI sequences

3 MRI scanners used for both MRI brain and MRI cervical spine:

1. 3T (Tesla) MR scanner (Achieva; Philips Healthcare, Netherlands) using an 8-channel SENSE head coil
2. 1.5T MR scanner (Ingenia; Philips Healthcare, Netherlands) using a 15-channel SENSE head coil
3. 1.5T MR scanner (Signa HDxt, General Electric Healthcare, Milwaukee, WI, USA) using an 8-channel brain coil

MRI brain diffusion weighted images (DWI) were acquired with the following parameters: repetition time (TR), 3155 milliseconds (ms); echo time (TE), 58.9 ms; flip angle, 90°; matrix, 124 × 124; section thickness, 4 millimetres (mm); 28 slices; axial acquisition; scan time, 1 minute (min) 50 seconds (s); b value, 1000s/mm^2^ on the Achieva; TR, 3455 ms; TE, 90.7 ms; flip angle, 90°; matrix, 112 × 109; section thickness, 5 mm; 28 slices; axial acquisition, scan time, 1 min 10 s; b value, 1000s/mm^2^ on the Ingenia; TR, 8000 ms; TE, 73.6 ms; flip angle, 90°; matrix, 128 × 128; section thickness, 5 mm; 28 slices; axial acquisition, scan time, 1 min 40 s; b value, 1000s/mm^2^ on the Signa HDxt.

MRI cervical spine studies included the following sequences: T2 sagittal, T1 sagittal, short tau inversion recovery (STIR) sagittal, T2 axial, gradient echo (GRE) axial. T2 sagittal images were acquired with the following parameters: TR, 3000 ms; TE, 100 ms; flip angle, 90°; matrix, 308 × 315; section thickness, 3 mm; 12 slices; scan time, 2 min on the Achieva; TR, 3000 ms; TE, 110 ms; flip angle, 90°; matrix, 416 × 254; section thickness, 3 mm; 12 slices; scan time, 2 min 30 s on the Ingenia; TR, 3000 ms; TE, 100 ms; flip angle, 90°; matrix, 320 × 260; section thickness, 3 mm; 12 slices; scan time, 2 min 42 s on the Signa HDxt. T1 sagittal images were acquired with the following parameters: TR, 715 ms; TE, 10 ms; flip angle, 90°; matrix, 300 × 300; section thickness, 3 mm; 12 slices; scan time, 2 min 45 s on the Achieva; TR, 488 ms; TE, 10 ms; flip angle, 90°; matrix, 356 × 251; section thickness, 3 mm; 12 slices; scan time, 3 min 11 s on the Ingenia; TR, 400 ms; TE, 9 ms; flip angle, 90°; matrix, 320 × 224; section thickness, 3 mm; 12 slices; scan time, 2 min 7 s on the Signa HDxt. STIR sagittal images were acquired with the following parameters: TR, 3554 ms, inversion time (TI), 200 ms; TE, 70 ms; flip angle, 90°; matrix, 252 × 200; section thickness, 3 mm; 12 slices; scan time, 3 min 33 s on the Achieva; TR, 2500 ms, inversion time (TI), 150 ms; TE, 50 ms; flip angle, 90°; matrix, 256 × 211; section thickness, 3 mm; 12 slices; scan time, 2 min 45 s on the Ingenia; TR, 3000 ms, inversion time (TI), 150 ms; TE, 28 ms; flip angle, 90°; matrix, 256 × 192; section thickness, 3 mm; 12 slices; scan time, 3 min 42 s on the Signa HDxt. T2 axial images were acquired with the following parameters: TR, 3000 ms; TE, 100 ms; flip angle, 90°; matrix, 248 × 200; section thickness, 4 mm; 30 slices; scan time, 4 min 33 s on the Achieva; TR, 3587 ms; TE, 100 ms; flip angle, 90°; matrix, 228 × 195; section thickness, 4 mm; 30 slices; scan time, 3 min 57 s on the Ingenia; TR, 6720 ms; TE, 93 ms; flip angle, 90°; matrix, 320 × 192; section thickness, 4 mm; 30 slices; scan time, 4 min 36 s on the Signa HDxt. GRE axial images were acquired with the following parameters: TR, 29 ms; TE, 7 ms; flip angle, 7°; matrix, 208 × 208; section thickness, 3 mm; 35 slices; scan time, 3 min on the Achieva; TR, 594 ms; TE, 6 ms; flip angle, 25°; matrix, 252 × 250; section thickness, 4 mm; 30 slices; scan time, 4 min 8 s on the Ingenia; TR, 1070 ms; TE, 16 ms; flip angle, 25°; matrix, 256 × 192; section thickness, 4 mm; 30 slices; scan time, 3 min 34 s on the Signa HDxt.
